# Estimating Potential Distribution of Sweetgum Pest *Acanthotomicus suncei* and Potential Economic Losses in Nursery Stock and Urban Areas in China

**DOI:** 10.3390/insects12020155

**Published:** 2021-02-11

**Authors:** You Li, Yuxuan Wan, Wei Lin, A. Simon Ernstsons, Lei Gao

**Affiliations:** 1 Vector-Borne Virus Research Center, Fujian Province Key Laboratory of Plant Virology, Fujian Agriculture and Forestry University, Fuzhou 350002, China; 2 State Key Laboratory for Ecological Pest Control of Fujian and Taiwan Crops, Fujian Agriculture and Forestry University, Fuzhou 350002, China; 3 School of Forest Resources and Conservation, University of Florida, Gainesville, FL 32611, USA; wanyuxuan@ufl.edu (Y.W.); aernstsons@ufl.edu (A.S.E.); 4 Chinese Academy of Forestry, Beijing 100091, China; 5 Technical Center of Gongbei Customs District P. R. China, Zhuhai 519001, China; gbhg5439@customs.gov.cn; 6 Shanghai Academy of Landscape Architecture Science and Planning, Key Laboratory of National Forestry and Grassland Administration on Ecological Landscaping of Challenging Urban Sites, Shanghai 200232, China

**Keywords:** *Liquidambar*, market price, removal cost, landscape, trap

## Abstract

**Simple Summary:**

American sweetgum *Liquidambar styraciflua* (Altingiaceae) was introduced to China over 60 years ago. It is an important tree species for increasing landscape value and promoting afforestation in urbanized areas of eastern China in the past 20 years. Sweetgum inscriber *Acanthotomicus suncei* is a local bark beetle pest and lethal to the introduced American sweetgum. To estimate the potential economic losses caused by the sweetgum inscriber, we survey the beetles’ natural distribution in China. Based on field collections, potential distribution was predicted. Nurseries stocking American sweetgum were found in the high suitability area of this pest. Additionally, we predict the direct loss incorporating tree and removal cost. A questionnaire was sent to maintenance companies, growers, and gardener associations for tree removal prices. In addition, the market price and inventory were also gained from telephone interview. Our economic analysis indicates that if sweetgum inscriber were to become established in the main American sweetgum business, the potential losses of nursery stock and urban area could range from USD 12.81 to 14.41 million. The results will serve as a baseline measure to control urban forest pests.

**Abstract:**

American sweetgum *Liquidambar styraciflua* (Altingiaceae) was first introduced to China over 60 years ago. It is an important tree species for increasing landscape value and promoting afforestation in urbanized areas of eastern China in the past 20 years. Sweetgum inscriber *Acanthotomicus suncei* (Coleoptera: Curculionidae) is a local bark beetle pest and lethal to the introduced American sweetgum. To provide preliminary estimates of the potential economic losses caused by the sweetgum inscriber, a field investigation was conducted to survey the beetles’ natural distribution in China. Based on field collections, potential distribution was predicted using Maxent. All nurseries stocking American sweetgum were in the high and very high suitability area of sweetgum inscriber. Additionally, we employed a model of direct loss incorporating tree and removal cost. A questionnaire was sent to maintenance companies, growers, and gardener associations for tree removal prices. We estimated the removal cost of each tree. In addition, the market price and inventory were also gained from telephone interview. Our economic analysis indicates that if sweetgum inscriber were to become established in the main American sweetgum business, the potential losses of nursery stock and urban area could range from USD 12.81 to 14.41 million.

## 1. Introduction

American sweetgum *Liquidambar styraciflua* (Altingiaceae) is native to North America and Mesoamerica [1]. About 60 years ago it was introduced to China and is considered an important alternative tree species for increasing landscape value and promoting afforestation in eastern urbanized areas in the past 20 years [2,3]. Furthermore, some cities nominate it for recommended urban tree species [4]. Traditionally, after an introduced ornamental tree species has demonstrated successful establishment in a new environment, including being noninvasive to indigenous plants, it will be broadly popularized. However, during this initial establishment, a local bark beetle pest in China, the sweetgum inscriber beetle (hereafter, SI) *Acanthotomicus suncei* Cognato (Coleoptera: Curculionidae), was identified causing damage to this tree species [5]. 

Sweetgum inscriber, a phloem-feeding beetle native to China, was only recently described to science. However, it has a history of damaging native sweetgum trees in China since 1959 [6,7]. In recent years, increased awareness of SI and ongoing survey efforts have led to the detection of numerous SI populations in urban and nursery areas [8]. Investigations indicated that more than 30,000 sweetgum trees (Diameter at breast height (DBH)= 5–25 cm) in Shanghai had been killed by SI up to 2018 [5,6], while no effective management has been developed. Heavy damage occurs negatively influencing the plant nursery stock in Shanghai and adjacent areas [5,9], while many American sweetgum were propagated by a limited number of nursery growers who were planning to sell these trees commercially. Furthermore, SI is often lethal to the American sweetgum. Meanwhile, SI is similar to other wood borer species where infested trees typically exhibit few or no external symptoms at low densities, or have symptoms easily confused with other tree pathogens [10,11,12]. Previous studies have focused on the emergence and developmental characteristics of SI adults in Shanghai as well as the fungi associated with SI in the field [8]. The infestations and identity of SI are rarely confirmed before tree mortality or wilt occurs [6]. Moreover, due to the transportation of nursery trees, there is high risk of SI dispersal to other areas. When SI has been recorded, all hosts were from urban parks or nurseries. It is still unknown where the natural distribution of SI is in Asia.

In response to the threat posed by SI in Eastern China, local agencies conduct surveys to detect new infestations, and support the research of SI ecology and management. The beetle also prompted international concern from the susceptible tree’s country of origin. Susaeta et al. estimates the potential economic losses to US plantation owners could be over USD 150 million, with higher ecological losses if SI is accidentally introduced to North America [13]. However, this US model is incompatible with the American sweetgum business in China. American sweetgum in China is a completely commercial product and only grown in nurseries before being sold, while in North America it grows widely in the field as pioneer tree species.

As other well-studied forest pests, assessing the potential economic impacts of SI is critical for evaluating the benefits of measures to decrease the damage to nursery stock and slow the range expansion. It will also support the continued promotion of American sweetgum and help develop restrictions of SI by pest control. Yet, there is little information on analog investments of urban forest pest management that are comparable with economic losses from SI damage, especially in developing countries. To help address this gap, we initially aimed to investigate the natural distribution of SI. Once known, we could predict the potential distribution of SI in China and determine how many American sweetgum nurseries were within the range of risk. We estimate the potential direct economic losses (cost of dead sweetgum) and indirect economic losses (cost of tree removal) by simulating continuation of SI infestation in its original distribution. We then calculate the market value of American sweetgum in urban areas and additional costs associated with dead tree treatment.

## 2. Materials and Methods

### 2.1. General Survey and Log Trapping for SI in China

In spring, summer and fall of 2017–2020, survey locations were centered on the known natural distribution of sweetgum tree *Liquidambar* spp. in China, because the current known host plant range of SI is limited to sweetgum trees [5]. At least one location of natural forest was selected in each province within the natural distribution of sweetgum tree in China. A general survey was initially conducted by inspecting the bark of sweetgum trees in the field and checking the bark beetle specimen collections from museums. In the field, natural fallen logs, recently damaged branches, or weakened trees were our main search focus as SI appear to prefer those wood materials. Log traps were also set when time and logging permits were available during the survey and established by cutting or girdling sweetgum tree logs or branches with diameter >10 cm. Wherever possible, logs were set in shaded forest areas to avoid sunlight exposure. Traps were checked after between 3 and 6 months. As SI was initially found in urban street trees, city parks and nurseries, we also conducted an additional survey in the urban area of some cities where American sweetgum is established. As there are few other bark beetle species that could infest sweetgum tree in the field, the presence of SI was only verified by adults. If suspected bark beetle adults from sweetgum trees were found, then samples were stored in pure alcohol for further identification in the lab. All bark beetle samples were sent to Forest Entomology Laboratory, University of Florida, USA or Institute of Plant Protection, Shanghai Academy of Landscape Architecture Science and Planning (SALASP), Shanghai, China for identification. All suspected samples were compared with a paratype and specimens were preserved with alcohol at −20 °C and stored at the SALASP and Forest Entomology, University of Florida. The confirmed occurrences of SI will be referred to as the geographical parameter of potential distribution (Section 2.2).

### 2.2. Potential Distribution Analysis

#### 2.2.1. Environmental Variables and Species Data

**Figure 1:**
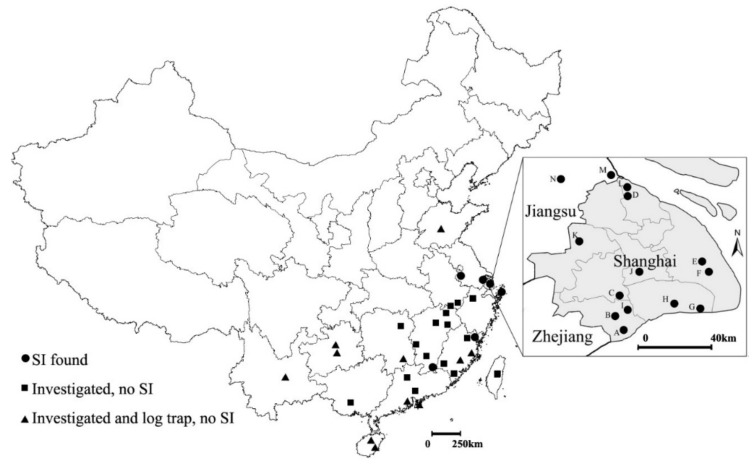
Distribution of SI in China; in the map of Shanghai (right), A–G indicate nurseries surveyed before 2016 [6]; H–N indicate new infestation sites in this study; grey area: Shanghai. SI: sweetgum inscriber.

Distribution data of SI were obtained from field investigation. Sixteen records were assembled (Figure 1). We used the receiver operating characteristic (ROC) and the area under the receiver operating characteristic curve (AUC) to evaluate the prediction results of the model [14].

Bioclimatic variables are crucial in defining species’ environmental niches. Data of 19 bioclimatic variables were downloaded from World Climate Database (worldclim.org), which provides the average data from 1970 to 2000, the spatial resolutions are 5 min. 

#### 2.2.2. Modeling Method and Statistical Analysis

MaxEnt is one of the mostly frequently used software packages to predict the suitability for spatial occurrence of a species by machine-learning algorithm [15]. It is confirmed to be relatively robust using small numbers of occurrence records [16]. Thus, MaxEnt (version 3.4.1) [17] was selected for the analysis of the potential distribution of SI. Nineteen environmental variables were implemented in the initial model (Table S1). As multiple collinearity may exist among the environmental variables, the variables were screened according to the percentage contribution and Pearson correlation coefficients between any two variables (Table S2) [18]. We removed the factors for which the absolute value of Pearson’s correlation coefficient is greater than or equal to 0.8 from the Pearson correlation coefficient table. Additionally, we considered the biological characteristics of SI. Therefore, minimum temperature of the coldest month (Bio 06) was kept, and temperature seasonality (standard deviation * 100) (Bio 04) was abandoned. Because Bio 04 was strong correlation with Bio 06 and Bio 06 has a greater impact on its biology [8]. Six environmental variables were chosen for SI (Table S2): Precipitation of the warmest quarter (Bio 18), Precipitation of the driest quarter (Bio 17), Precipitation seasonality (coefficient of variation) (Bio 15), Precipitation of the driest month (Bio 14), Mean temperature of the warmest quarter (Bio 10) and Bio 06 in the models of MaxEnt. In the final model, ‘Create response curves’ and ‘Do jackknife to measure variable importance’ were chosen, Output format ‘Logistic’ was chosen, the ‘Replicates’ was set as 10, the ‘Replicated run type’ was chosen as ‘Crossvalidate’. Other parameters in the MaxEnt model were kept as default. The results of the model were predicted with the area under the curve (AUC) using the receiver operator characteristic. As some research demonstrated, the fixed threshold approach is not optimal [19,20,21], thus the MaxEnt results here were imported into ArcGIS 10.4.1 and reclassified into five categories (Very Low, Low, Medium, High, Very High) representing the suitability values using Jenks Natural Breaks Classification [22,23]. The prediction result will determine if the urban area or nursery will be under the likely SI area of effect.

### 2.3. Potential Economic Loss in China

Estimating the potential economic loss of nursery and urban forestry stock is a complicated issue requiring knowledge of organismal ecology, species interaction, and known distribution. Here, two major expenses, (1) direct cost: market value; (2) indirect cost: tree removal cost, were analyzed. The potential economic loss (*L*) of nurseries in other areas accounting for the average local price (*P*), inventories (*I*), and tree removal cost (*Cpt*) of American sweetgum in each nursery is:

(1) $$L\;=\;\overset{n}{\underset{n=1}{\sum }}\left(P_{n}+Cpt\right)\times I_{n}\times M$$

where *M =* 84.30% represents mortality of sweetgum tree by SI from previous study [5]. The information provided by nursery growers and the current market price of American sweetgum in different diameter at breast height (DBH) were acquired from the biggest Chinese landscape website Zhongguoyuanlin (yuanlin.com) and largest retailer platform Taobao (taobao.com). Plant inventory data of each nursery grower were reported via telephone. Even though prices of different DBH are available, and some inventories provide larger trees (DBH > 8cm), we are estimating the loss with the average price of sweetgum trees at DBH = 8 cm for several reasons: (1) to account for price and inventory fluctuations. (2) due to trade secrets, the specific number of larger size sweetgums (DBH > 8cm) are not accessible from some respondents (nurseries or companies). (3) The inventories of American sweetgum with DBH ≥ 8 cm from all respondents are complete and available. (4) These trees usually have the minimum landscape value and are also suggested as the most appropriate size (DBH ≥ 8 cm) of street tree planted in urban areas by the local Bureau of Landscape and Forestry in China (DG/TJ08-53-2016) [24]. 

The tree removal cost survey instrument consists of questionnaires to tree maintenance companies in the nursery and urban landscape industries. The local municipal Bureau of Landscape and Forestry in China usually maintains the urban forest through publicly tendered hiring specialization of maintenance companies. Therefore, the target population for this survey was forestry maintenance companies across eastern China likely to apply for tendered contracts. The sample expense was the removal cost of a dead or diseased sweetgum tree. The underlying assumption was that if sweetgum trees are damaged and killed by SI, removal cost in urban area and nursery is a significant component. The sampling frames for each city were records of American sweetgum introduced and planted along the street or urban park. Contacts for tree maintenance company were provided by SALASP which is the first organization to introduce and establish American sweetgum tree in China in urban forestry settings.

Generally, the tree removal costs mainly consist of four parts, cutting, digging up roots, loading and transport. For most companies, the costs of destroying the diseased or infected tree would be included in carriage costs. Assuming *Cc, Cr, Cl*, and *Ct* denote the cost of cutting (*Cc*), digging up roots (*Cr*), loading (*Cl*), and transport (*Ct*), respectively. Therefore, the tree removal costs C can be expressed as:

(2) $$Cpt\;=\;\frac{Cc\;+\;Cr\;+\;Cl\;+\;Ct}{\mathrm{number}\;\mathrm{of}\;\mathrm{trees}\;\mathrm{removed}\;}$$

where *Cc* and *Cr*, form the budget quota of landscape project in China, and could be collected from Glodon Digital Platform (www.glodon.com). The damage and loss in Shanghai are excluded from this study as there are relatively few sweetgum trees that survived in Shanghai after SI outbreaks between 2013 and 2018. Most sweetgum trees were cut in Shanghai prior to 2018.

## 3. Results

### 3.1. Original Distribution

A total of 38 sites were investigated between March 2017 and March 2020 (Figure 1). Log traps were set in 19 field sites during the survey (Figure 1, Table S3). In Shanghai, since the first investigation of SI damage in nurseries (Figure 1: Spots A–G) before 2017 [6], seven additional locations, five in Shanghai and two in adjacent Jiangsu Province, were also observed under attack by SI (Figure 1: Spots H–N). Outbreaks (where most planted sweetgum in the sites are killed) were also observed on ornamental sweetgums planted outside of the nurseries in Jiangsu (Figure 1, M and N). Nanjing is also included as type material of SI was from there. Except for Shanghai and Jiangsu, SI were also found in a EtOH trap in Zhoushan, Zhejiang Province, and Chinese sweetgum *Liquidambar formosana* in Fu’an, Fujian Province and Ganzhou, Jiangxi Province (Figure 1). The three new collections were not from nursery or ornamental trees. No outbreaks were observed in the field. In Ganzhou, Jiangxi Province, we also discovered SI could live on *L.* (*Altingia*) *gracilipes*. The infestation is sporadic and nearby trees (<50 m) were inspected for signs of attack, but no symptoms were observed.

### 3.2. Potential Distribution

**Figure 2:**
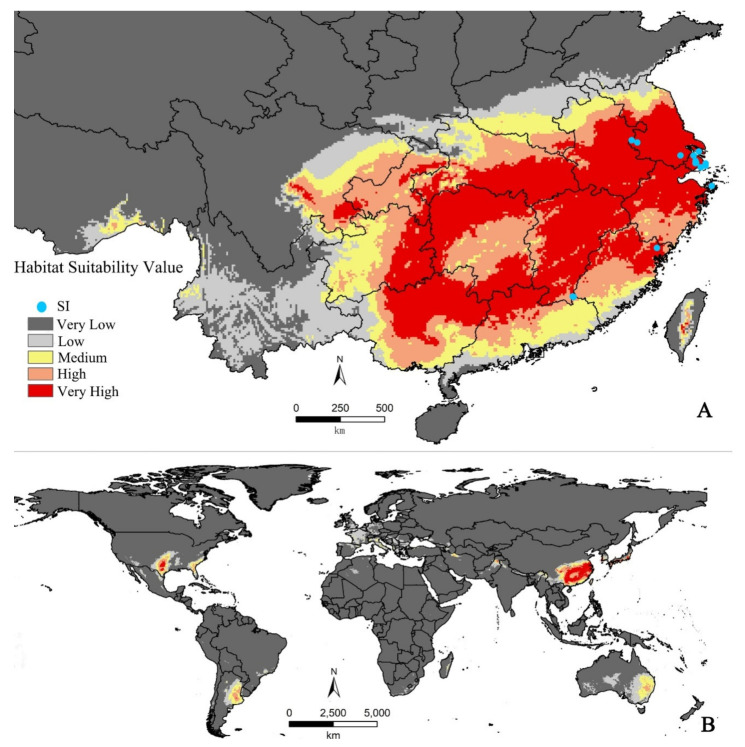
Potential geographical distribution of SI by MaxEnt; (**A**) potential distribution in China; (**B**) potential world distribution. SI: sweetgum inscriber.

The model predicted that the climatically suitable areas of SI lie mainly in subtropical humid monsoon, mid-latitude monsoon, and temperate continental climate zones (Figure 2). In China, high and medium suitable habitat suitability areas under current climate scenarios were located in Central Taiwan, Shanghai, Zhejiang, Jiangsu, Fujian, Anhui, Jiangxi, Guangdong, Hubei, South Henan, Hunan, Guangxi, Guizhou, Chongqing, and East Sichuan (Figure 2A).

**Figure 3:**
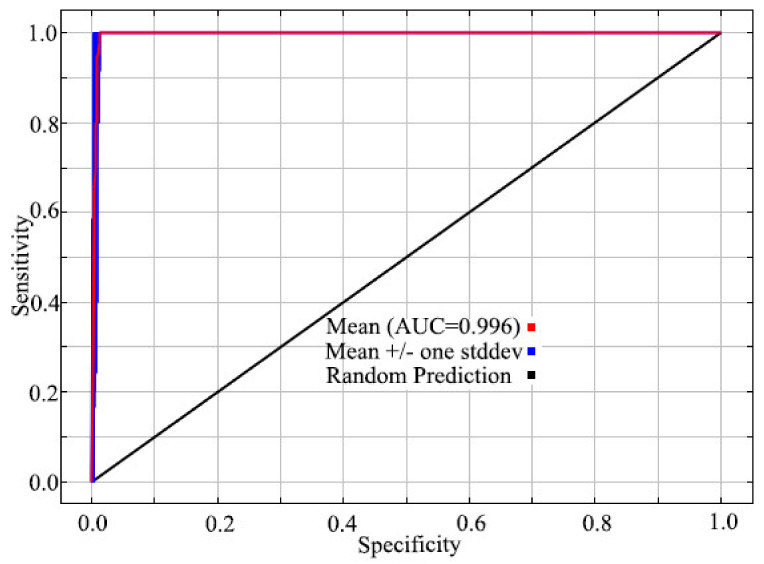
ROC curve and AUC values for current MaxEnt model. ROC: receiver operating characteristic. AUC: the area under the receiver operating characteristic curve.

Under current climate scenarios for SI, the MaxEnt model performed reasonably well having an AUC value of 0.996 with a standard deviation of 0.003 (Figure 3). Minimum temperature of the coldest month (Bio 06) was the best predictor. The second leading was Precipitation of the warmest quarter (Bio 18) (Table 1, Figure S1). 

### 3.3. Nurseries or Landscape Company Own American Sweetgum in China

**Figure 4:**
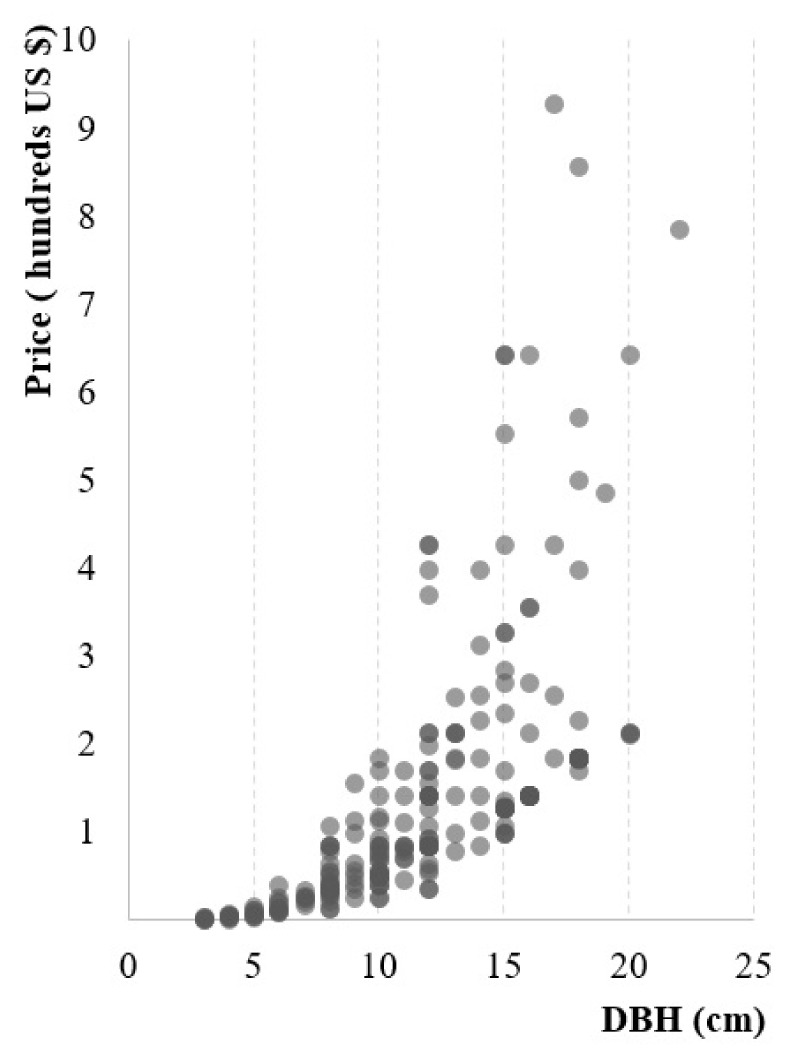
The market price of American sweetgum in different DBH at 2019–2020. DBH: diameter at breast height.

In total, 52 nurseries or landscape companies distribute American sweetgum based on internet information searches. Forty-two companies provided quotations with prices of different DBHs (Figure 4). Following interview by telephone survey, 32 companies were confirmed with inventories of American sweetgum with DBH ≥ 8 cm (Table S4). Those nurseries were distributed in six provinces (Table 2) and all located in the high or very high risk areas in the potential distribution of SI (Figure 2). The range value of inventories of American sweetgum with DBH ≥ 8 cm of each company and province was also provided (Table 2, Table S4). In total, 68.75% surveyed nurseries were from Jiangsu province. The average market price of American sweetgum DBH = 8 cm in each province ranged from USD 37.14 to 64.29.

### 3.4. Cost of Tree Removal in China

Ten nurseries or landscaping companies completed the questionnaires for tree removal (Table 3, Tables S5 and S6). The cutting costs (*Cc*) for an arbor tree 20 ≤ DBH < 50 cm is between USD 3.0 and 9.1. The costs to remove roots are the same. For loading cost (*Cl*), it includes cost of instrument (excavator, crane) and labor. According to the survey, the price of a 25t crane is around USD 157.14–228.57 per day and the price of a 20t excavator is around USD 214.29–357.14 per day. The average daily cost of labor is USD 22.86 per person, and the average number of laborers is nine per day, however, the exact number should depend on tree size and number. Although the average rental price of a trailer was calculated, which is USD 57.14 each time, it is difficult to estimate *Ct* since the time depends on number and size of trees. Thereby, we assumed that one trailer transports 20 trees of DBH ≤ 20 cm or 15 trees of 20–30 cm DBH each time to calculate *Ct*. Due to the short establishment time in the region, it is rare to see larger size (DBH>20 cm) American sweetgum in the investigation. Therefore, we only choose the lower estimated *Cpt* of USD 16.01 for Section 3.5.

### 3.5. Potential Economic Loss in China

We estimated the loss of market value with the minimum value size DBH = 8 cm. This was due to frequent changes in market price, sweetgum tree growth speed, and SI preference of attacking trees with DBH of specific size (DBH = 5–25 cm) [6]. Jiangsu province will suffer the most serious loss about USD 9.67–10.88 million if a widespread SI outbreak were to occur. Across other regions in the potential distribution of SI, the total estimate of potential economic loss by SI will reach USD 12.81–14.41 million in China.

## 4. Discussion

The prediction of SI distribution is based on the result of large-scale field investigation. Increasing the number of survey sites would improve the accuracy of the prediction. Although there is only sparse data of SI in natural vegetation [8], we still successfully recorded it in a general survey and log traps in Fujian and Jiangxi. However, due to limited manpower or expenditure, there are still some areas at the southern and northern edges where the sweetgum tree naturally grow that were not surveyed. Additionally, as SI is tiny and cryptic in the field, only trained or experienced bark beetle experts could find them successfully. Further research is needed that may find further populations in other regions of China or other countries.

Like other *Acanthotomicus* spp., it appears to have an observable host-selection habit [25]. Wood and Bright [26] recorded that different *Acanthotomicus* species may have significantly different host plants, but most of them are usually specific to plants in one genus or family. In addition, if introduced plants are systematically closely related to native plants, local pests of native plants generally will eventually feed on exotic plants [27] which was observed in previous studies. Therefore, SI attacking fresh cut indigenous *Liquidambar* spp. is not unexpected. 

The prediction is in line with natural distribution of local sweetgum trees. Nursery grower and horticultural company introductions of American sweetgum align with the distribution of local sweetgum species. Therefore, all surveyed nursery companies were within the potential distribution area. It indicates those companies may suffer losses where attacks of SI take place, as they did previously in Shanghai. The Maxent prediction also including North America, the native continent of American sweetgum is also within the suitability area of SI (Figure 2). In particular, four southern US states, Florida, Georgia, Texas, and Oklahoma, were in the risk showing high and very high suitable areas, while American sweetgum also commonly grows there [28]. Our result could also contribute to the test of global sentinel gardens as sweetgum is an introduced ornamental plant in many countries and widely planted outside of its native range [29]. 

The two infestations of SI found in southern China were only from log traps. This indicates SI is likely playing the same ecological role as other bark beetles within the Ipini tribe in the natural environment [30,31,32]. Similarly, these outbreaks of bark beetles could occur when subjected to a change of environment. Managed trees, such as those in nursery or urban settings are subjected to different environmental stressors compared to more natural settings. These stressors accelerate regional environmental change and may predispose trees to attack. As all trees are likely in a uniform condition, when SI is found in a nursery, the most common result is all sweetgum are abandoned after chemical pest control measures fail.

Due to knowledge gaps in SI ecology and habit, we could not investigate or estimate the mortality in a base line situation. As shown in Table 2, we referred to the known mortality in nurseries, rather than providing different scenarios to predict the effect of management. Similar methods are commonly used in economic impact models in forest pest and disease management [13,33]. The range values of our final estimate were produced using the inventory information. The quotations from nursery and landscape companies only provided a range of stock with DBH ≥ 8 cm. Some nurseries actually own many bigger trees but exact quantities of different sizes were not public. Therefore, the estimate from the price of DBH = 8 cm could be undervalued (Figure 4). As an ornamental tree species, the chief value of American sweetgum in urban area is the visual colored leaf [34]. Its market price is dramatically increased after trees mature and develop larger canopies [35]. Over time, those prices and values will change.

The estimate of cost (direct and indirect) was among the weaknesses in our model. Compared to other economic tree species, especially for conifer species in plantation forests and fruit tree species in orchards, sweetgum is substantially small in China. Therefore, the sample size may be insufficient and price fluctuation is difficult to predict. However, we warn against overinterpretation of our prediction. Our results did not include policy interference or administrative cost. For example, local departments recommended other urban tree species due to the concern of SI [4,36]. Many other non-artificial factors could also affect the analysis. As such, we likely produced lower bound estimates with less elements involved. Expected damages of a new pest or a naïve plant are complicated as they rely on several assumptions [37,38]. In this study, those assumptions included. (1) the current actual distribution of SI is not disturbed by human influence. Either in China or other countries, the actual distribution can be affected by human influence. Fortunately, the current known actual distribution of SI is limited to China. (2) All American sweetgum nurseries will likely encounter this pest due to its known distribution, and that pest management is ineffective. Strict quarantine during transportation and delivery will inhibit the spread of SI between nurseries in different areas. Prompt eradication after detection is difficult but may reduce the loss [5].

Landscape value is the main benefit of American sweetgum stock in China. After breeding in nurseries, they will be planted in urban areas, such as parks, streets, and suburban environments. This establishment is comparatively expensive. When SI is introduced in these situations, the cost will also largely increase. The removal of undesirable damaged trees is in addition to the cost of replacement. We formulated a model to determine the removal costs depending on tree size based on the current Chinese market (see Equation (2)). Given our past experience and interviews with nursery growers, the removal costs of small-size trees are lower than that of large-size due to increasing complexity, and costs of time and labor. It is possible that a similar situation would be observed in other pests or trees. We added the larger tree (DBH > 20 cm) followed by our main objective small size tree (DBH ≤ 20 cm) to provide more references. Those removal costs are also flexible from an economic perspective, because in China, local forestry authorities have to select an executing company by public bidding. Additionally, the prices of excavator use, cranes, labor, and trailers are different in different areas and subject to seasonal demand.

## 5. Conclusions

Our field investigation confirmed that SI is naturally distributed in southern China and parasitic on weakened indigenous *Liquidambar* spp. in natural vegetation. It is likely SI could occupy most areas surrounding surveyed nurseries who distribute American sweetgum. Our economic analysis indicates that if SI were to become established in the main American sweetgum business, the losses could range from USD 12.81 to 14.41 million. Notwithstanding, the impact on the local nursery or landscape business could be catastrophic as many of these commercial ornamental trees were transported to different areas. Many other nurseries or companies not covered in our survey may also suffer financial loss. It is imperative that efforts be made to impose quarantines to restrict the trans-regional movement of American sweetgum in China and restrict the spread of SI. The spread of SI may pose a threat to other countries and the potential damage could be more deleterious than implied in this study. The model we used could also focus on other pest–plant interactions in urban areas of eastern China. 

## Figures and Tables

**Table 1:** Estimates of relative contributions of the environmental variables to the MaxEnt model.

Variable Number	Variable Name	Percent Contribution	Permutation Importance
bio_06	Min temperature of the coldest month	55.2	59.4
bio_10	Mean temperature of the warmest quarter	3.6	11.2
bio_14	Precipitation of the driest month	7.4	21.4
bio_15	Precipitation seasonality	4.9	1
bio_17	Precipitation of the driest quarter	5.4	5.9
bio_18	Precipitation of the warmest quarter	23.5	1.2

**Table 2:** Estimate of economic loss (*L*) under impacts of SI on sweetgum in the nursery of China (currency unit: USD).

Province	Number of Nurseries in the Survey	Inventories of American Sweetgum with DBH ≥ 8 cm (*I*)	Average Local Price of American Sweetgum with DBH = 8 cm (*P*)	Estimate of Economic Losses (*L*; million USD)
Anhui	1	15,000–16,000	45.71	0.78–0.83
Henan	1	10,000–11,000	37.14	0.45–0.49
Hunan	1	7000–8000	64.29	0.47–0.54
Jiangsu	22	188,200–211,700	44.94	9.67–10.88
Sichuan	3	10,500–13,500	53.33	0.61–0.79
Zhejiang	4	14,000–15,000	53.57	0.82–0.88
In total	32	244,700–275,200		12.81–14.41

**Table 3:** Survey of tree removal at eastern China in 2018–2019, separated by tree DBH (currency unit: USD).

Urban Tree Size	Number of Maintenance Company in the Survey	Total Number of Tree Removal	Average				Cost Per Tree (*Cpt*)
			*Cc*	*Cr*	*Cl*	*Ct*	
Small size (DBH ≤ 20 cm)	10	1040	312.00	312.00	683.71	309.29	16.01
Large size (20 < DBH < 30 cm)	4	230	525.71	525.71	803.21	264.29	37.05

## Data Availability

The data presented in this study are openly available in supplementary material and Mendeley Data, doi:10.17632/rr5kwfb98f.1.

## Supplementary Materials

The following are available online at https://www.mdpi.com/2075-4450/12/2/155/s1, Table S1: Percent contribution of all variables used in the MaxEnt analysis. Table S2: Pearson correlation of all variables used in the MaxEnt analysis. Table S3: Field survey result from 2017 to 2020. Table S4: Information from each nursery in this study. Table S5: Questionnaire result of removal costs for sweetgum tree (DBH ≤ 20 cm). Table S6: Questionnaire result of removal costs for sweetgum tree (20 < DBH < 30 cm). Figure S1: Jackknife test for variable importance in the SI suitability distribution: values shown are averages over 10 replicate runs.
