# Supplementary material for: Estimating Potential Distribution of Sweetgum Pest Acanthotomicus suncei and Potential Economic Losses in Nursery Stock and Urban Areas in China
Source: Insects. 2021 Feb 11;12(2):155. doi: 10.3390/insects12020155 (PMC7918347; doi:10.3390/insects12020155)
Supplement: Supplementary file 1 [file insects-12-00155-s001.pdf]

Supplementary Material

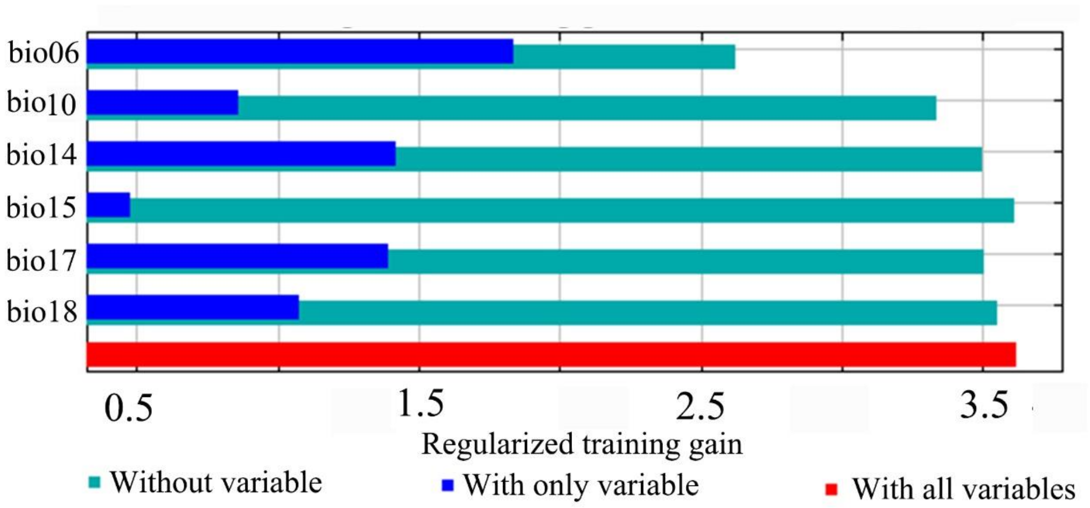

Figure S1. Jackknife test for variable importance in the SI suitability distribution: values shown are averages over 10 replicate runs.

Table S1 Percent contribution of all variables used in the MaxEnt analysis.

Variable	Percent contribution
bio_18	20.7
bio_17	20.6
bio_04	14.1
bio_08	11.5
bio_15	9.2
bio_14	8.6
bio_06	4.4
bio_13	3.2
bio_02	2.9
bio_10	1.7
bio_03	1.1
bio_11	1
bio_16	0.5
bio_05	0.2
bio_09	0.1
bio_01	0
bio_12	0
bio_19	0
bio_07	0

Table S2 Pearson correlation of all variables used in the MaxEnt analysis.

Pearson correlation	bio1	bio_2	bio_3	bio_4	bio_5	bio_6	bio_7	bio_8	bio_9	bio_10	bio_11	bio_12	bio_13	bio_14	bio_15	bio_16	bio_17	bio_18	bio_19
bio1	1	.443	.860**	-.857**	-.364	.884**	-.785**	-.650**	.666**	-.426	.941**	.783**	.721**	.263	.340	.780**	.565*	.690**	.793**
bio_2	.443	1	.692**	-.258	.188	.130	-.041	-.330	-.011	-.040	.294	.397	.631**	-.446	.889**	.599*	-.205	.657**	.120
bio_3	.860**	.692**	1	-.875**	-.482	.788**	-.748**	-.841**	.551*	-.641**	.875**	.899**	.931**	.027	.665**	.950**	.414	.899**	.755**
bio_4	-.857**	-.258	-.875**	1	.762**	-.973**	.975**	.911**	-.751**	.828**	-.978**	-.941**	-.822**	-.361	-.284	-.869**	-.702**	-.761**	-.938**
bio_5	-.364	.188	-.482	.762**	1	-.693**	.836**	.781**	-.551*	.934**	-.633**	-.748**	-.586*	-.345	-.012	-.603*	-.706**	-.532*	-.767**
**bio_6**	.884**	.130	.788**	-.973**	-.693**	1	-.975**	-.823**	.780**	-.734**	.983**	.861**	.699**	.465	.119	.760**	.731**	.626**	.924**
bio_7	-.785**	-.041	-.748**	.975**	.836**	-.975**	1	.867**	-.764**	.847**	-.944**	-.886**	-.713**	-.461	-.095	-.765**	-.774**	-.641**	-.940**
bio_8	-.650**	-.330	-.841**	.911**	.781**	-.823**	.867**	1	-.618*	.900**	-.833**	-.946**	-.889**	-.204	-.430	-.885**	-.568*	-.802**	-.846**
bio_9	.666**	-.011	.551*	-.751**	-.551*	.780**	-.764**	-.618*	1	-.571*	.761**	.628**	.440	.482	.025	.549*	.598*	.445	.809**
**bio_10**	-.426	-.040	-.641**	.828**	.934**	-.734**	.847**	.900**	-.571*	1	-.694**	-.811**	-.693**	-.286	-.208	-.713**	-.577*	-.627**	-.769**
bio_11	.941**	.294	.875**	-.978**	-.633**	.983**	-.944**	-.833**	.761**	-.694**	1	.900**	.784**	.375	.266	.839**	.691**	.727**	.921**
bio_12	.783**	.397	.899**	-.941**	-.748**	.861**	-.886**	-.946**	.628**	-.811**	.900**	1	.945**	.245	.464	.958**	.700**	.908**	.913**
bio_13	.721**	.631**	.931**	-.822**	-.586*	.699**	-.713**	-.889**	.440	-.693**	.784**	.945**	1	-.027	.696**	.974**	.478	.959**	.753**
**bio_14**	.263	-.446	.027	-.361	-.345	.465	-.461	-.204	.482	-.286	.375	.245	-.027	1	-.580*	.051	.754**	.016	.563*
**bio_15**	.340	.889**	.665**	-.284	-.012	.119	-.095	-.430	.025	-.208	.266	.464	.696**	-.580*	1	.683**	-.204	.733**	.129
bio_16	.780**	.599*	.950**	-.869**	-.603*	.760**	-.765**	-.885**	.549*	-.713**	.839**	.958**	.974**	.051	.683**	1	.529*	.973**	.799**
**bio_17**	.565*	-.205	.414	-.702**	-.706**	.731**	-.774**	-.568*	.598*	-.577*	.691**	.700**	.478	.754**	-.204	.529*	1	.504*	.869**
**bio_18**	.690**	.657**	.899**	-.761**	-.532*	.626**	-.641**	-.802**	.445	-.627**	.727**	.908**	.959**	.016	.733**	.973**	.504*	1	.729**
bio_19	.793**	.120	.755**	-.938**	-.767**	.924**	-.940**	-.846**	.809**	-.769**	.921**	.913**	.753**	.563*	.129	.799**	.869**	.729**	1
****. Correlation is significant at the 0.01 level (2-tailed).**																			
***. Correlation is significant at the 0.05 level (2-tailed).**																			

Table S3. Field survey result from 2017 to 2020

Location no.	Province	Nearest city	Longitude (N) and latitude (E)	Investigate method	Found SI
1	Fujian	Fu'an	27.160250, 119.686083	log trap and search	Yes
2	Jiangsu	Taicang	31.591694, 121.075889	search	Yes
3	Jiangsu	Nanjing	31.990583, 118.775528	search, old specimens, and literature	Yes
4	Jiangxi	Xunwu	24.924472, 115.829556	log trap and search	Yes
5	Zhejiang	Zhoushan	29.978694, 122.268111	unknown trap	Yes
6	Jiangsu	Suzhou	31.406386, 120.7545	search	Yes
7	Shanghai	Shanghai	31.034505, 121.9023833	log trap and search	Yes
8	Shanghai	Shanghai	31.05116, 121.43838	log trap and search	Yes
9	Shanghai	Shanghai	31.5892, 121.5943055	log trap and search	Yes
10	Shanghai	Shanghai	31.400425, 121.351991	log trap and search	Yes
11	Shanghai	Shanghai	30.93006, 121.897608	log trap and search	Yes
12	Anhui	Huangshan	30.100739, 118.201575	log trap and search	No
13	Anhui	Yaoli	29.558541, 117.606260	log trap and search	No
14	Fujian	Yongchun	25.315323, 118.288535	log trap and search	No
15	Fujian	Fuzhou	25.817612, 119.134105	log trap and search	No
16	Fujian	Ningde	27.160416, 119.503412	log trap and search	No
17	Guangdong	Guangzhou	23.178105, 113.366475	search	No
18	Guangdong	Nanling	24.731043, 112.962617	search	No
19	Guangdong	Zhuhai	22.304698, 113.569524	log trap and search	No
20	Guangxi	Shangsi	21.879045, 107.939673	log trap and search	No
21	Guizhou	Guiyang	26.554866, 106.752126	log trap	No
22	Guizhou	Xiuwen	26.845396, 106.578021	log trap	No
23	Hainan	Danzhou	19.511351, 109.472744	log trap	No
24	Hainan	Wanning	18.803733, 110.237112	log trap	No
25	Hong Kong	Kadoorie center	22.429561, 114.113729	log trap and search	No
26	Hunan	Chenzhou	25.749167, 112.808297	log trap	No
27	Hunan	Changsha	28.188312, 112.939035	search	No
28	Jiangxi	Jingdezhen	29.409781, 117.339786	log trap	No
29	Jiangxi	Jiulianshan	24.930431, 113.095816	search	No
30	Jiangxi	Jinggangshan	26.684878, 114.160895	search	No
31	Jiangxi	Yiyang	28.321069, 117.400364	search	No
32	Jiangxi	Wancunzhen	29.604273, 117.748498	search	No
33	Shandong	Taian	36.158011, 117.069265	log trap and search	No
34	Taiwan	Nantou	24.070775, 120.998629	search	No
35	Yunnan	Kunming	25.136874, 102.744304	log trap	No
36	Zhejiang	Changhua	30.084690, 118.852401	search	No
37	Fujian	Yunxiao	23.952512, 117.312640	search	No
38	Fujian	Wuping	25.181209, 116.135259	search	No

Table S4. Information from each nursery in this study (Currency unit: US dollar $)

Nursery	Province	Price of American sweetgum (DBH=8 cm)	Inventories of American sweetgum (DBH≥8 cm)
SC1	Sichuan	51.43	500
SC2	Sichuan	68.57	3000–5000
SC3	Sichuan	40.00	7000–8000
AH1	Anhui	45.71	15000
HEN1	Henan	37.14	10000
HUN2	Hunan	64.29	7000–8000
ZJ1	Zhejiang	61.43	1000–1000
ZJ2	Zhejiang	64.29	3000–4000
ZJ3	Zhejiang	42.86	5000
ZJ4	Zhejiang	45.71	5000
JS1	Jiangsu	57.14	1500
JS2	Jiangsu	37.14	200
JS3	Jiangsu	50.00	1500–2000
JS4	Jiangsu	37.14	10000–20000
JS5	Jiangsu	85.71	1000
JS6	Jiangsu	37.14	1000
JS7	Jiangsu	50.00	1000
JS8	Jiangsu	34.29	1000–2000
JS9	Jiangsu	40.00	20000
JS10	Jiangsu	45.71	3000–5000
JS11	Jiangsu	37.14	3000–5000
JS12	Jiangsu	50.00	3000–5000
JS13	Jiangsu	37.14	3000–5000
JS14	Jiangsu	37.14	50000
JS15	Jiangsu	37.14	50000
JS16	Jiangsu	54.29	5000
JS17	Jiangsu	37.14	5000
JS18	Jiangsu	28.57	5000
JS19	Jiangsu	37.14	5000
JS20	Jiangsu	37.14	5000
JS21	Jiangsu	107.14	7000–10000
JS22	Jiangsu	14.29	7000–8000

Table S5. Questionnaire result of removal costs for sweetgum tree (DBH≤20 cm) [Currency unit: US dollar $; *Cc*, *Cr*, *Cl* and *Ct* indicate the cost of cutting (*Cc*), digging up roots (*Cr*), loading (*Cl*) and transport (*Ct*)]

Company	Number of tree removal	*Cc*	*Cr*	*Cl*	*Ct*	Cost per tree (*Cpt*)
Company 1	150	450.00	450.00	480.00	457.14	12.29
Company 2	150	450.00	450.00	708.57	514.29	14.14
Company 3	80	240.00	240.00	900.00	342.86	21.57
Company 4	80	240.00	240.00	521.43	285.71	16.14
Company 5	80	240.00	240.00	642.86	171.43	16.14
Company 6	100	300.00	300.00	614.29	214.29	14.29
Company 7	100	300.00	300.00	650.00	357.14	16.14
Company 8	120	360.00	360.00	657.14	257.14	13.57
Company 9	80	240.00	240.00	757.14	171.43	17.57
Company 10	100	300.00	300.00	905.71	321.43	18.29
Average		312	312	683.71	309.29	16.01

Table S6. Questionnaire result of removal costs for sweetgum tree (20<DBH<30 cm) [Currency unit: US dollar $; *Cc*, *Cr*, *Cl* and *Ct* indicate the cost of cutting (*Cc*), digging up roots (*Cr*), loading (*Cl*) and transport (*Ct*)]

Company	Number of trees removed	*Cc*	*Cr*	*Cl*	*Ct*	Cost per tree (*Cpt*)
Company 3	60	548.57	548.57	900.00	342.86	39.00
Company 7	60	548.57	548.57	650.00	285.71	33.88
Company 9	60	548.57	548.57	757.14	171.43	33.76
Company 10	50	457.14	457.14	905.71	257.14	41.54
Average		525.71	525.71	803.21	264.29	37.05
